# Investigation of the influence of a glutathione S-transferase metabolic resistance to pyrethroids/DDT on mating competitiveness in males of the African malaria vector, *Anopheles funestus*

**DOI:** 10.12688/wellcomeopenres.15013.2

**Published:** 2019-03-21

**Authors:** Magellan Tchouakui, Billy Tene Fossog, Brigitte Vanessa Ngannang, Doumani Djonabaye, Williams Tchapga, Flobert Njiokou, Charles S. Wondji

**Affiliations:** 1 Department of Animal Biology and Physiology, Faculty of Science, University of Yaoundé 1, Yaoundé, P.O. Box 812, Cameroon; 2 Department of Medical Entomology, Centre for Research in Infectious Diseases, Younde, 13591, Cameroon; 3 Department of Biochemistry, University of Yaoundé 1, Yaoundé, P.O. Box 812, Cameroon; 4 Department of Vector Biology, Liverpool School of Tropical Medicine,, Pembroke Place, L35QA, Liverpool, UK, UK

**Keywords:** Malaria, insecticides, metabolic resistance, Glutathione S-transferase, Anopheles funestus, mating competitiveness

## Abstract

**Background:** Metabolic resistance is a serious challenge to current insecticide-based interventions. The extent to which it affects natural populations of mosquitoes including their reproduction ability remains uncharacterised. Here, we investigated the potential impact of the glutathione S-transferase L119F-GSTe2 resistance on the mating competitiveness of male *Anopheles funestus*, in Cameroon.

**Methods: **Swarms and indoor resting collections took place in March, 2018 in Tibati, Cameroon. WHO tube and cone assays were performed on F _1 _mosquitoes from indoor collected females to assess the susceptibility profile of malaria vectors. Mosquitoes mated and unmated males collected in the swarms were genotyped for the L119F metabolic marker to assess its association with mating male competitiveness.

**Results**: Susceptibility and synergist assays, showed that this population was multiple resistant to pyrethroids, DDT and carbamates, likely driven by metabolic resistance mechanisms. Cone assays revealed a reduced efficacy of standard pyrethroid-nets (Olyset and PermaNet 2.0) with low mortality (<25%) whereas synergist PBO-Nets (Olyset Plus and PermaNet 3.0) retained greater efficacy with higher mortality (>80%). The L119F-GSTe2 mutation, conferring pyrethroid/DDT resistance, was detected in this *An. funestus* population at a frequency of 28.8%. In addition, a total of 15 mating swarms were identified and 21 *An. funestus* couples were isolated from those swarms.  A comparative genotyping of the L119F-GSTe2 mutation between mated and unmated males revealed that heterozygote males 119L/F-RS were less able to mate than homozygote susceptible (OR=7.2, P<0.0001). Surprisingly, heterozygote mosquitoes were also less able to mate than homozygote resistant (OR=4.2, P=0.010) suggesting the presence of a heterozygote disadvantage effect. Overall, mosquitoes bearing the L119-S susceptible allele were significantly more able to mate than those with 119F-R resistant allele (OR=2.1, P=0.03).

**Conclusion:** This study provides preliminary evidences that metabolic resistance potentially exerts a fitness cost on mating competiveness in resistant mosquitoes.

## Background

Despite significant reduction of malaria burden in the past decade, this disease remains a major public health concern in Africa. Recent reports of increase cases of malaria ^1^ is a further indication that more is needed to control this disease. The scale up of vector control measures, in particular long-lasting insecticide treated nets (LLINs) and indoor residual spraying (IRS), has been the main driver of this reduction of malaria burden with about 78% of all gains achieved since 2000 attributed to these methods ^2^. However, resistance is spreading in malaria vectors in Africa including *Anopheles funestus* for the four classes of insecticides used in public health, compromising the effectiveness of these interventions ^3^. Providing adequate information about the mechanisms of resistance and more importantly its impact on key traits of mosquito biology, ecology and behaviour such as their mating ability in the presence of resistance can help in planning and implementing suitable insecticide resistance management strategies.

Insecticide resistance management strategies including rotation of insecticide rely on the assumption that insecticide resistance alleles are very often detrimental in the absence of insecticide selection pressure ^4, 5^. The adaptive allele in this case might be associated with modification of physiological processes or resource availability ^6^ which can lead to decreased performance and fitness disadvantage of resistant mosquitoes ^7– 9^ and therefore, a reversal to susceptibility is expected in the absence of selection pressure from the specific insecticide. However, little is currently known on such fitness costs in field populations of malaria vectors notably for metabolic resistance mechanism because of a lack of DNA-based markers. A previous study using a laboratory strains of *An. gambiae*, demonstrated fewer copulations in dieldrin resistant males when compared with their susceptible counterparts ^6^. Berticat *et al.* demonstrated also the disadvantage in competitive mating ability of *Culex pipiens* males with the target-site resistance *Ace1R* genotype, when compared with susceptible males, pointing to its potential impact on the spread and persistence of resistant alleles. In contrast, for malathion resistance in the beetle *Tribolium castaneum* it was noticed that resistance enhanced male reproductive success. If this last case is observed in malaria vectors, it will be a great concern for control program as it will prevent the implementation of resistance management strategies based on the rotation of insecticides. Currently, there is little information on the impact of metabolic insecticide resistance on the mating ability of natural populations of major malaria vectors in Africa. So far the only study on this topic reported a lack of impact of metabolic resistance on male competitiveness of *An. gambiae* field population in Burkina Faso ^10^. The study assessed only the global transcription profiling of mated and unmated mosquitoes since a lack of DNA marker for metabolic resistance prevented a direct genotyping correlation with mating status. However, recent progress made in elucidating the molecular basis of metabolic resistance had identified a single amino acid change (L119F) in the glutathione S-transferase epsilon 2 (GSTe2) conferring pyrethroid/DDT resistance in *An. funestus* ^11^. This new marker now provide the opportunity to directly investigate the impact of metabolic resistance on mating male competitiveness. However, assessing the impact of resistance on the mating of malaria vectors through swarm’s collection in natural populations of mosquitoes requires a good knowledge of the mating places and also the mating behaviour of these vectors.

Concerning the mating behaviour of insects, it was reported that most of them mate in swarms, whereby dispersed populations aggregate at specific times and places ^12, 13^. In mosquitoes including malaria vectors, swarming occur very often around visual markers such as vegetation and brick piles on the ground ^14– 16^. This knowledge on mating places and behaviour can also help to reduce mosquito densities or interrupt the mating thus helping to reduce pathogen transmission in vector populations ^17^. This technique has been effective against some Anopheles mosquitoes in Burkina Faso but on a limited scale ^18^. Little information is currently available for other vector species like *An. funestus*. In *An. gambiae* s.s. mating is limited to a very short period at dusk. In this species, males always swarming before and disbanding after copulation ^19^. Females approach a swarm, promptly acquire a male and leave in copula ^19, 20^. Mating behavior, which is one of the most important aspects of reproduction ^21^, remains widely under-investigated in many malaria vectors. While many studies were conducted on *An. gambiae* swarms in Western Africa ^10, 20, 22, 23^, observations have rarely been reported in Eastern, Southern and Central Africa. Prior to this current report, there has been little information available on the swarms in Cameroon. Improved understanding of mosquito mating systems, and more importantly how insecticide resistance mechanisms affects the mating success in field population of malaria vectors such as *An. funestus,* could possibly give new tools for vector control implementation.

In this study, after characterizing the mating swarms of *An. funestus*, we investigated the resistance profiling and molecular basis of insecticide resistance in a natural populations of *An. funestus* in Cameroon. Furthermore, we investigated the potential impact of metabolic resistance on mating male competitiveness by assessing the association between the L119F-GSTe2 metabolic resistance marker and the mating success of *An. funestus* mosquitoes in field conditions.

## Methods

### Study area

Initially, the surveys covered two villages, Tibati (6°28’ N, 12°37’ E) and Mibellon (6°46’N, 11° 70’E) (Adamawa Region, Cameroon) but we eventually focused on just one village (Tibati) according to the density of the swarms. The main malaria vectors in Tibati are *An. funestus* during the dry season and *An. gambiae* s.l during the rainy season, whereas in Mibellon *An. funestus* is the predominant species ^24^. The dominance of *An. funestus* in these areas is due to the presence of multiple lakes known as suitable breeding sites for this species. LLINs is the main vector control approach in Cameroon. The villages included in this study benefited from universal LLIN distribution in 2011 and 2016. Because of high selection pressure of insecticide contained in the LLINs, the main malaria vectors have developed resistance to pyrethroids used in the nets ^25^. The communities rely mainly on substance farming, agriculture but also fishing.

### Detection and collection of *An. funestus* swarms

Swarm collections were undertaken on 12 evenings in February and March 2018. The search for potential swarms in each village started in the first evening at sunset around 5.30 pm, and then each swarm located were characterized and/or collected throughout the study. Swarms were searched in various places (around the potential breeding sites, closer to habitations, the farms, on the street) with the presence of potential markers assessed. For all swarms identified, different characteristics such as i) heights ii) starting time of swarming, iii) time at night when the swarms became invisible and iv) the behaviour of mosquitoes in the swarms were recorded. Swarms were then sampled using sweep nets as described previously ^10, 20, 23^. All couples of *An. funestus* (mated) were extracted from the swarms and each couple manually transferred into a clean cartoon cup. Samples of the remaining males that did not mate in the same swarms were collected. All mosquitoes sampled were separated into unmated males, mated males and mated females and stored in RNA-later solution for further analysis.

### Indoor female collections and F _1_ rearing

For the purpose of assessing the susceptibility profile to various public health insecticides and WHO recommended bed nets, F _1_ females were generated from indoor-resting blood-fed (F _0_) females collected using electric aspirators. Collected mosquitoes were morphologically identified using the key of Gillies and De Meillon (1968). After sampling, female mosquitoes were transferred to the insectary of the Centre of Research in Infectious diseases (CRID) in Yaoundé, Cameroon. Female mosquitoes collected were kept in standard insectary conditions of 25 ± 2°C, 80 ± 10% relative humidity and fed with 10% sugar solution for at least four days and then left to oviposit using the forced-egg laying method ^26^. F _1_ larvae were reared to adults using the protocol previously described ^26^.

### Species identification

Genomic DNA was extracted from 40 F _0_ *An. gambiae* s.l. and 102 F _0_ *An. funestus* s.l female mosquitoes (head and thorax) using the Livak protocol ^27^ which includes grinding of mosquito in a Livak buffer, followed by a 65°C incubation for 30 min and then centrifugation. Further steps involved an incubation on ice (30min) followed by centrifugation steps, precipitation with alcohol (100% and 70%) ^27^. Mosquito species was identified using the Koekomoer cocktail Polymerase Chain Reaction (PCR) assay for *An. funestus* group and the SINE PCR assay for *An. gambiae* s.l. ^28, 29^.

### Infection of malaria vectors by *Plasmodium* parasites

*Plasmodium* infection rate was estimated by Taqman (401400, Santa Clara, CA, USA) assay using the head and thorax of F _0_ field-collected mosquitoes as previously described ^30^. 102 females *An. funestus* sensu stricto (s.s.) and 40 *An. gambiae* s.l were used for the detection of *Plasmodium falciparum* (falcip+) and/or *Plasmodium ovale*, *Plasmodium vivax*, and *Plasmodium malariae* (OVM+) sporozoites.

### Insecticide susceptibility assays

Susceptibility profiles to insecticides using WHO bioassays were assessed using the F _1 _generation of *An. gambiae* s.l. and *An. funestus* s.s. according to WHO procedures ^31^. Insecticides tested for *An. funestus* included permethrin (0.75%) (PE 452), deltamethrin (0.05%) (DE 609), bendiocarb (0.1%) (BE 172), propoxur (0.1%), dichlorodiphenyltrichloroethane (DDT) (4%) (DD 226), malathion (5%) (MA 215), fenitrothion (1%) (FE 205) and dieldrin (4%) (DI 094) (VCRU, Penang, MALAYSIA). Due to a limited number of F _1_ *An.* gambiae s.l from field collected mosquitoes, only females were tested for DDT, permethrin and deltamethrin. Control mosquitoes were exposed to non-impregnated papers. The mortality rates were determined 24h post-exposure to insecticide. In addition to the 60 min exposure described above, mortality after 30 min, 90min, 2h and 3h exposures to DDT, deltamethrin and bendiocarb was also assessed in order to evaluate the intensity of resistance of *An. funestus* s.s from Tibati.

### Synergist assays

To assess the contribution of cytochrome P450 and GST enzymes in the resistance profile, synergist assays were performed with PBO (Piperonyl Butoxide) and DEM (Diethyl Maleate) with *An. funestus* s.s. Four replicates of 20–25 adult mosquitoes (2–5 day old) were immediately exposed to permethrin (0.75%), deltamethrin (0.05%), or DDT (4%) for 60 minutes after pre-exposed to PBO or DEM impregnated papers (4 %) for 1 hour. In addition, control assays using only the synergist impregnated papers for 60 minutes were also performed and mortality recorded 24 hours after. The mortality rate obtained were compared with those without synergist’s exposure using a chi square test.

### Assessment of bed net efficacy using cone assays

In order to assess the impact of resistance on insecticide-based interventions against the malaria vectors of this location, we checked the efficacy of common bed nets recommended by WHO against the Tibati’s *An. funestus* population. 3 minute cone bioassays were carried out following the WHO guidelines ^31^. Five batches of ten F _1_ females (2–5 days old) were placed in plastic cones attached to 5 commercial nets: PermaNet ^®^ 2.0 (deltamethrin 1.8 g/kg) (Vestergaard, Lausanne, Switzerland), PermaNet ^®^ 3.0 (side of the net; deltamethrin 2.8g/kg) (Vestergaard, Lausanne, Switzerland), PermaNet ^®^ 3.0 (top of the net; deltamethrin 4.0 g/kg plus 25g/kg piperonyl butoxide (PBO)) (Vestergaard, Lausanne, Switzerland), Olyset ^®^ (2 % permethrin) (Sumitomo Chemical UK PLC, London, UK) and Olyset ^®^ plus (2 % permethrin plus 1 % PBO) (Sumitomo Chemical UK PLC, London, UK).

### Genotyping of resistance marker and assessment of the impact on the mating male competitiveness of *An. funestus* field population

L119F-GSTe2 metabolic and A296S-RDL target-site resistance markers, involved in DDT/permethrin and dieldrin resistance in *An. funestus* respectively were genotyped in order to assess the effect of these resistance mechanisms on the mating ability of *An. funestus* field population as there is no evidence of *kdr* in *An. funestus* ^32^. The L119F-GSTe2 was genotyped using an allele-specific (AS)-PCR and the A296S-RDL by TaqMan assay (Santa Clara, CA, USA). A296S-RDL TaqMan reaction was performed as previously described ^33^ PCR reactions (10 μl) contained 1 μl of genomic DNA, 5μl of SensiMix DNA kit (catalog: SM2-717104), 0.125μl of the A296S-RDL probe and 3.875 μl of sigma water. Samples were run on a Mx3000P™ (Stratagene) using the temperature cycling conditions of: 10 minutes at 95°C followed by 40 cycles of 95°C for 10 seconds and 60°C for 45 seconds. We designed a new allele specific PCR to genotype the L119F-GSTe2 mutation ^9^. Two pairs of primers were used for the AS-PCR (two outer and two inner primers, Table 1). Specific primers were designed manually to match the mutation and an additional mismatched nucleotide was added in the 3 ^rd^ nucleotide from the 3′ end of each inner primer to enhance the specificity. PCR was carried out using 10 mM of each primer and 1ul of genomic DNA as template in 15 μl reactions containing 10X Kapa Taq buffer A (KB 1003), 0.2 mM dNTPs (DM-516404), 1.5 mM MgCl _2 _(KB 1001), 1U Kapa Taq (KE 1000) (Kapa biosystems). The cycle parameters were: 1 cycle at 95°C for 2 min; 30 cycles of 94°C for 30 s, 58°C for 30 s, 72°C for 1min and then a final extension at 72°C for 10 min. PCR products were separated on 2% agarose gel by electrophoresis.

**Table 1.** Details of primer sequences used to analyse the L119F *GSTe2* mutation.

Primers	Sequence (5’ to 3’)
Ndel_Gste2F	GGAATTCCATATGACCAAGCTAGTTCTGTACACGCT
Xbal_Gste2 R	TCTACATCAAGCTTTAGCATTTTCCTCCTT
L119F-Res	CGGGAATGTCCGATTTTCCGTAGAA **t**A **A**
L119-F-Sus	CATTTCTTATTCTCATTTACAGGAGCGTA **a**T **C**

Furthermore, in an effort to characterize the broad dynamic of resistance to insecticides in this location, we also genotyped the L1014F target-site knockdown resistance (Kdr w) associated with DDT/pyrethroid resistance in *An. gambiae* using a Taqman (Santa Clara, CA, USA) method as previously described ^34^. PCR reactions (10 μl) contained 1 μl of genomic DNA, 5μl of SensiMix DNA kit (catalog: SM2-717104), 0.125μl of the L1014F-kdrw probe and 3.875 μl of sigma water. Samples were run on a Mx3000P™ Multiplex quantitative PCR system (Stratagene) using the temperature cycling conditions of: 10 minutes at 95°C followed by 40 cycles of 95°C for 10 seconds and 60°C for 45 seconds.

### Statistical analysis

Association between the *GSTe2* mutation and mating success was assessed by calculating the odds ratio of mating between the homozygous resistant, heterozygous and susceptible for each gene in mated males compared to unmated group with statistical significance based on the Fisher’s exact probability test. All analyses were conducted using GraphPad Prism version 7.00.

## Results

### Mosquito composition at Tibati

A total of 1021 blood fed female of *An. funestus* s.l. were collected indoors. Molecular identification on 102 *An. funestus* s.l mosquitoes confirmed that they were all *An. funestus s.s*. Only 40 *An. gambiae s.l* were collected and molecular identification revealed that the majority was *An. gambiae* s.s at 82.5% (33/40) whereas 17.5% (7/40) were *Anopheles coluzzii*.

### *Plasmodium* infection rate

Out of the 102 *An. funestus* s.s tested by Taqman, 2.94% (3/102) mosquitoes were sporozoite infected with *P. falciparum.* Due to low sample size, *Plasmodium* infection rate in *An. gambiae* s.l was assessed in both species combined ( *An. gambiae* (n= 33) and *An. coluzzii* (n=7)). This revealed that 12.5% (5/40) *An. gambiae* s.l. mosquitoes were infected with sporozoites predominantly with *falciparum* (falcip+; 10% [4/40]), whereas one mosquito was *P. ovale/vivax/malariae* infected (OVM+; 2.5% [1/40]). Two out of the five infected *An. gambiae s.l.* were *An. coluzzii* [2/7 infected (28.5%)] and three were *An. gambiae* [3/33 infected 9.1%)]. However, the low sample size of *An. coluzzii* means that this rate is not comparable.

### Collection of the *An. funestus* swarms

15 swarms with considerably large size (more than 100 mosquitoes/swarm) were observed in Tibati, while very few swarms (with small size, less than 50 mosquitoes/swarm) were observed in Mibellon. Most mating swarms were located close to human habitations compared to other places and swarming started with two to three mosquitoes congregating after sunset around 6.05pm, and flying above a swarm place. The number of mosquitoes increased in the swarms over the next 5 to 10 minutes and slowly decreased in size then disappeared after 30 minutes when the sky became dark. Flying mosquitoes were observed by viewing them against the sky after sunset. Males of *An. funestus* swarmed at the height of 2.5m from the ground. Concerning the mating behaviour, when a female coupled with a male, they immediately left the swarms, flying at 1.5m from the ground. It is at that moment that the couples were extracted from the swarms using the sweep nets. There was no clear physical marker for *An. funestus* swarm’s position in Tibati but the commonest place for swarming were just an empty space close to habitations and most of the swarm locations remained the same for several days. Throughout this survey, we observed and collected a total of 21 copulation events in Tibati. Furthermore, we collected more than 1000 male mosquitoes from those remaining in the swarms after a mating period (that most likely did not mate). The low number of collected couples suggests a low number of females in these swarms but could also indicate that mating was also taking place in other swarms not detected in this study.

### Resistance profile of malaria vectors in Tibati

**Figure 1.**
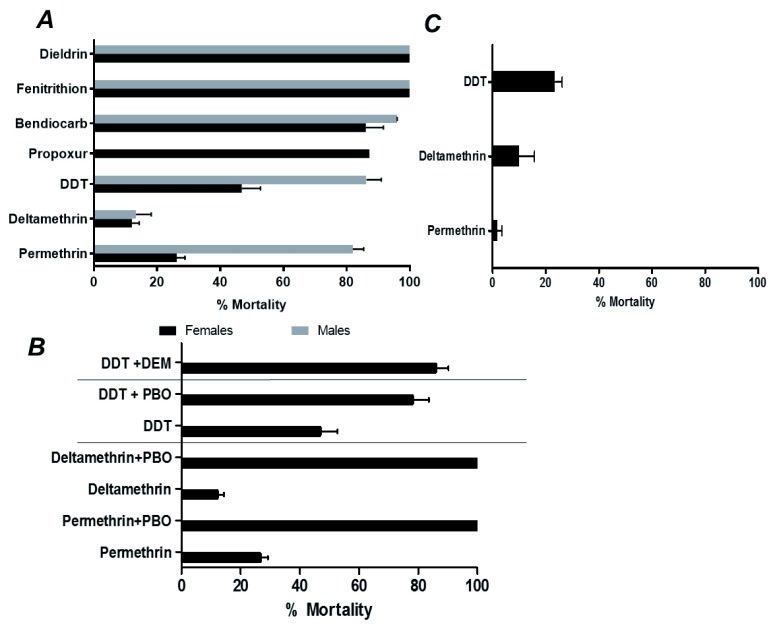
Susceptibility profile to main insecticides of malaria vectors in Tibati. ( **A**) Susceptibility profile of *Anopheles funestus sensu stricto* and ( **B**) susceptibility profile of *Anopheles funestus* s.s females after synergist assay with PBO and DEM whereas ( **C**) susceptibility profile of *Anopheles gambiae* sensus lato population. Error bars represent standard error of the mean. Abbreviations: DDT, dichlorodiphenyltrichloroethane; PBO, piperonyl butoxide; DEM, diethyl maleate.

**Figure 2.**
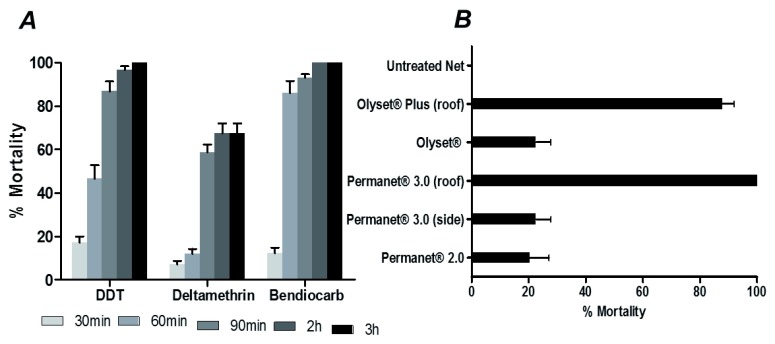
Exploration of resistance intensity in *An. funestus* and impact on LLINs. ( **A**) Susceptibility profile at different time point exposure to DDT, deltamethrin and bendiocarb. ( **B**) Bioefficacy of different commercial long-lasting insecticidal nets against *Anopheles funestus* s.s using cone assays. Error bars represent standard error of the mean.

*An. funestus* s.s exhibited full susceptibility to organophosphates (malathion and fenitrothion) and to dieldrin (organochlorine) with 100% mortality rate. This population showed high level of resistance to pyrethroids with low mortality rates in females including permethrin (type I; 26.6% ± 2.6 mortality) and deltamethrin (type II; 12.0% ± 2.3 mortality). Resistance was observed against the organochlorine DDT (46.8% ± 5.9 mortality), but only a moderate resistance was recorded against the carbamates bendiocarb (86.1% ± 5.5% mortality) and propoxur (87.2% ± 0.8 mortality) ( Figure 1A). The males also exhibited similar susceptibility patterns to the females ( Figure 1A). Due to the high resistance observed for pyrethroids and DDT insecticides, the intensity level of this resistance was assessed by performing bioassays with higher exposure times of 90min, 120min and 180min for deltamethrin and DDT, and also for bendiocarb ( Figure 2A). After 2h and 3h exposure to deltamethrin, mosquitoes still exhibited a mortality rate of <80% (2 hours: 67.4% ± 4.5; 3 hours: 76.4% ± 3.6). In contrast, mortality rates close to 100 were observed with DDT aft 2h and 3h exposure (2h: 96.7% ± 1.7; 3 h: 100% ± 00), and for bendiocarb (90 min: 93.1% ± 1.6; 2 hours: 100% ± 00).

 Analysis of *An. gambiae* s.l. mosquitoes revealed that this population was generally more resistant than *An. funestus* with lower mortality rates observed for DDT (23.6% ± 2.6 mortality), permethrin (1.75% ± 1.75) and deltamethrin (10.0% ± 5.8%) ( Figure 1C).

### Synergist assays

Synergist assays showed a full recovery to susceptibility after PBO pre-exposure for both type I and II pyrethroids tested (permethrin: no PBO pre-exposure (26.6% ± 2.6) mortality vs PBO pre-exposure [100.0% ± 0.0], X² = 73.9; P < 0.0001); deltamethrin: no PBO pre-exposure [12.0% ± 2.3%] vs PBO pre-exposure [100% ± 0.0], (X² = 107.30; P <0.0001)), suggesting that cytochrome P450 enzymes may be playing a major role in pyrethroid resistance in *An. funestus* s.s. from Tibati ( Figure 1B). Tests with DDT also revealed the impact of PBO pre-exposure although the susceptibility was not fully recovered (DDT: no PBO pre-exposure [46.78% ± 5.95%] vs PBO pre-exposure [78.1% ± 5.6%], X² = 13.4; P = 0.0003) suggesting that other gene families or mechanisms contribute to DDT resistance. For this reason, we assessed the implication of GSTs enzymes by performing a bioassay with 1h pre-exposition to DEM (inhibitors of GSTs). This revealed a recovery, although only partial (DDT: no DEM pre-exposure [46.8% ± 5.9%] vs DEM pre-exposure [85.9% ± 4.3%], (X² = 22.36; P <0.0001), showing that GSTs, probably GSTe2 ^11^, is contributing synergistically with cytochrome P450 enzymes to the resistance to DDT in this *An. funestus* population.

### Bio-efficacy of commercialized nets against *An. funestus* in Tibati

A low efficacy of standard nets (Olyset and PermaNet 2.0) was observed against *An. funestus* s.s.: Olyset: 22.6 ± 5.1% mortality, PermaNet 2.0: 20.4 ± 6.7%. In contrast PBO-based nets (OlysetPlus, and PermaNet 3.0) showed an increased efficacy (OlysetPlus: 87.9 ± 3.9% mortality; PermaNet 3.0-side: 64.2 ± 6.9%, PermaNet 3.0-roof: 100.0 ± 0.0%) ( Figure 2B).

### Frequency of knockdown resistance ( *kdr*) in *An. gambiae*

Taqman genotyping of L1014F target-site resistance mutation in *An. gambiae s.l* revealed that the frequency of 1014F kdr resistant allele was high (72.7% [48/66]) in Tibati in accordance with high pyrethroid and DDT resistance. 66.7% [22/33] were homozygote resistant, 12.1% [4/33] heterozygote whereas 21.2% [7/33] were homozygote susceptible.

### Genotyping of L119F-GSTe2 metabolic resistance and impact on the mating success of *An. funestus s.s* field population

**Figure 3.**
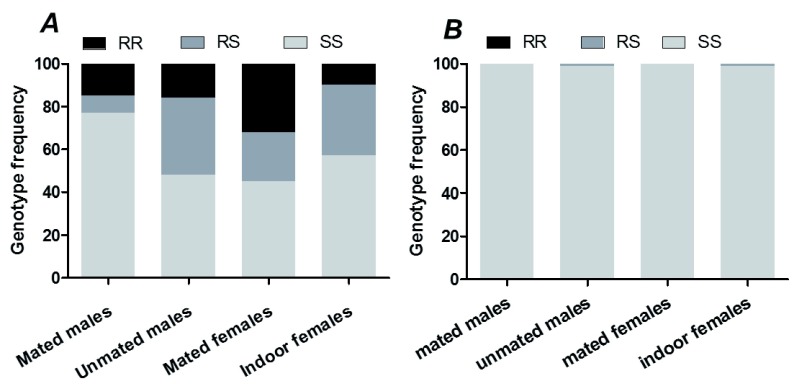
Distribution of resistance markers in *An. funestus* in Tibati between coupled males, uncoupled males and coupled females. ( **A**) L119F-GSTe2 genotypes and ( **B**) A296S-RDL genotypes.

Genotyping of L119F-GSTe2 mutation in indoor collected females revealed a frequency of 28.8%, comprising 10.2% (13/127) 119F/F-RR homozygous resistant, 33.1% (42/127) 119L/F-RS heterozygotes and 56.7% (72/127) L/L119-SS homozygous susceptible ( Table 2; Figure 3A). Moderate frequency of the 119F resistant allele in all samples, was recorded in mated (23.8%) compared to unmated males (33.5%) ( Table 2). Direct comparison of the frequency of each genotype between mated and unmated males revealed no significant differences between all groups of mosquitoes (P≥ 0.16). However, an assessment of the association of each genotype with mating success using odds ratio (OR) revealed that the heterozygote genotype (L119F-RS) showed a significantly lower chance of mating than both homozygous resistant (OR = 4.2 IC: 1.49- 11.9; P< 0.01) and homozygous susceptible mosquitoes (OR = 7.2 IC: 3.1 - 16.8; P < 0.0001) ( Table 3; Figure 3A). In contrast, no significant difference was observed between Homozygote resistant and homozygote susceptible mosquitoes (OR=1.77; IC 0.77-3.7; P=0.22). The impact of the resistant allele 119F on the mating competitiveness was also supported by the significantly greater likelihood of not mating when possessing this resistant allele than the susceptible L119 (OR=2.1; CI 1.1-4.0; P=0.03) ( Table 4).

**Table 2.** Distribution of L119F-GSTe2 genotypes between mated males, mated females and unmated males compared to indoor collected females.

	Genotypes		
Phenotypes	119F/F-RR	119L/F-RS	L/L119-SS
Mated males	4 (19%)	2 (10%)	15 (71%)
Unmated males	14 (16%)	33 (36%)	44 (48%)
Mated females	7 (33%)	4 (19%)	10 (48%)
Indoor females	13 (10%)	42 (33%)	72 (57%)
*Allele*	119F		L119
Mated	23.8%		76.2%
Unmated males	33.5%	/	66.5%
Mated females	42.9%		57.1%
Indoor females	26.8%		73.2%

**Table 3.** Distribution of A296S-RDL between mated males, mated females and unmated males compared to indoor collected females.

	Genotypes		
Phenotypes	296S/S-RR	A296S -RS	A/A296 -SS
Mated males	0	0	21
Unmated males	0	1	95
Mated females	0	0	17
Indoor females	0	1	126
*Allele*	296S		A296
Mated	0%		100%
Unmated males	0.52%	/	99.48%
Mated females	0%		100%
Indoor females	0.40%		99.60%

**Table 4.** Assessment of the association of different genotypes at L119F-GSTe2 mutation with mating success; *, significant difference.

Genotypes	*L119F-GSTe2*	
	Odds ratio	P-value
**SS vs RR**	**1.77** (0.77– 3.77)	0.22
**SS vs RS**	**7.2** (3.1 – 16.8)	<0.0001 *
**RR vs RS**	**4.2** (1.49-11.9)	0.010 *
**S vs R**	**2.1** **(1.1-4.0)**	0.03 *

### Genotyping of A296S-RDL target-site resistance in *An. funestus s.s*

Genotyping of A296S-RDL mutation associated with dieldrin resistance in mated and unmated males revealed that the 296S resistant allele is almost absent in this location ( Table 2, Figure 3B). These results were confirmed by the full susceptibility observed with dieldrin in the bioassay test. For this reason, no further comparison was performed for this mutation about its impact on mating success.

## Discussion

Elucidating the malaria vector ecology and behaviour is crucial for the implementation of alternative control measures in order to achieve the aim of malaria elimination. Mating is one component of mosquito behaviour that remains poorly characterized. After characterizing an *An. funestus* population in Cameroon including insecticide resistance profiling and swarm patterns, we took advantage of the recent detection of the glutathione S-transferase L119F-GSTe2 marker in *An. funestus* ^11^ to investigate the potential influence of metabolic resistance on mating competitiveness of male *An. funestus* mosquitoes.

### Species composition and their contribution to malaria transmission in Tibati

*An. funestus s.s* was the dominant vector in during the study coinciding with the dry season where the presence of large and permanent breeding sites as the lakes and the rivers facilitate the proliferation of this species contrary to *An. gambiae s.l* ^35^. A contrasted sporozoite infection rate between both species was noticeable with high rates in *An. gambiae s.l* (12.5%), but low for *An. funestus s.s* (2.9%). The significant difference between the two species is not commonly seen in Cameroon ^35, 36^ or DR Congo ^37^, as both species tend to present similar infection rates. It could be that the difference observed here is due to the ecological dynamic between the two species as it is possible that due to favorable conditions for *An. funestus*, there is an expansion of the populations of this species with more young individuals, whereas *An. gambiae* s.l population is made of older individuals in which the *Plasmodium* parasite has already completed its full extrinsic cycle since collection was done during the dry season.

### High level of insecticide resistance in malaria vectors in Tibati

This study revealed a high level of resistance to multiple insecticide classes in *An. funestus s.s* and *An. gambiae s.l* which, together with their high level of *Plasmodium* infection rate, calls for urgent actions to be taken to control malaria in this region as in Cameroon. Both malaria vectors were highly resistant to pyrethroids, the only insecticide class recommended for bed nets ^3^. *An. gambiae* were also found to be resistant to pyrethroids and DDT. This resistance profile is similar to that observed in Cameroon ^25, 38^, and in Central Africa as recently reported in DR Congo ^37^. Similar observations were also reported in Kenya, Madagascar, Tanzania and Uganda ^39– 42^ where this species was highly resistant to these insecticides. The Tibati *An. funestus* population was also resistant to pyrethroids and DDT, almost at the same level as *An. gambiae*. *An. funestus* mosquitoes showed some level of resistance to carbamates: bendiocarb and propoxur similar to reports in Northern Cameroon ^43^. The common used Olyset and Permanet 2.0 LLINs presented a very low bioefficacy against *An. funestus* in cone assays. The low efficacy of this two nets, treated with permethrin and deltamethrin only, is wide-spread in *An. funestus* populations across the continent ^33, 37, 44^. This loss of efficacy of these pyrethroid-only nets correlates well with the very high permethrin and deltamethrin resistance observed for this species. However, the greater efficacy with PBO-based nets (OlysetPlus and PermaNet 3.0) possibly provides an alternative solution to control this species for which resistance is mainly metabolic with an important role played by cytochrome P450 as shown by the synergist PBO assay. However, the spread or increased frequency of GSTe2-mediated resistance could limit the efficacy of such PBO-nets in the future. This is supported by the only partial recovery of susceptibility observed with (Olyset Plus), coupled with the increased mortality with the DEM synergist assay. The impact of GST-mediated resistance on efficacy of PBO-based nets will need to be assessed particularly in situations where such mechanism become predominant, as reported in Benin ^11, 45^.

### Swarming habits and behaviour of *An. funestus*

We observed in both Mibellon and Tibati that the heights of swarms were around 2.5m from the ground. This is in line with findings of Charlwood *et al.* in Mozambique ^46^, and Zawada in Zambia ^47^ where they noticed that *An. funestus* swarmed 2–4m from the ground. However, Harper in one study observed that *An. funestus* swarms occur immediately inside the threshold of a hut, and swarming occurred a foot off the ground ^48^. Since molecular analysis were not conducted in the study of Harper, it’s possible that mosquitoes he collected in the swarms was another member of the *An. funestus* group. There is also the possibility that depending on environmental conditions, *An. funestus* have changed its swarming behaviour and position. However, future studies in other locations are required to address this variation in *An. funestus* mating behaviour.

Swarming behaviour of *An. funestus* in this study was also different to that reported for *An. gambiae*. It is reported that members of the *An. gambiae* complex swarm around markers such as brick piles, rice fields, banana trees, burnt ground, garbage heaps and ant hills ^49, 50^, however, *An. funestus* swarms we observed in this study appeared to avoid ground markers . As observed in Nchelenge, Zambia ^47^, there was no clear physical marker for *An. funestus* swarm’s position in Tibati, but the most common place for swarming were empty spaces close to habitations, and most of the swarm locations remained the same for several days. This supports the suggestion of Charlwood *et al.* that mosquitoes of *An. gambiae* complex and *An. funestus* have different swarm markers.

As reported in other studies ^12, 50^, mosquito swarms in Tibati occurred perpetually in the same locations at approximately the same time each day. This phenomenon needs to be assessed in other parts of Africa, which may allow the swarm to be targeted as an alternative control measure for malaria prevention. It is also unknown if *An. funestus* mate in fewer large swarms or in multiple small swarms. The number of mosquitoes in swarms as reported by Charlwood *et al.* ^46^ were also relatively low, and on average less than 50 adults/swarm in Mibellon. In contrast, as reported by Harper ^48^, about 300–500 mosquitoes were present in each swarm in Tibati during the collecting period.

### Association between GSTe2-mediated metabolic resistance and mating success of *An. funestus*

This study revealed a negative impact of L119F-GSTe2 DDT/pyrethroid resistance on the mating competitiveness of males *An. funestus* as possessing the 119F resistant allele reduced the likelihood of mating. This is the first report of such negative impact of metabolic resistance on the mating success of field malaria vectors. The reduced fitness of L119F resistant mosquitoes observed in this study may suggests that the L119F mutation in the *GSTe2* gene potentially affects some physiological traits in resistant mosquitoes including mobility, perception of stimuli or even the olfactory system as the target site resistance ^4, 5^. However, heterozygote mosquitoes were more affected by this negative impact than homozygote resistant individuals suggesting a heterozygote disadvantage effect. In contrast, the study conducted in Vallée du Kou in Burkina Faso on the male of *An. coluzzii* mosquitoes reported a heterozygote advantage for the target site resistance mechanisms. It was observed that *kdr* heterozygote males were more likely to mate than homozygote resistant counterparts and heterozygote RDL _R_/RDL _S _were also more likely to mate than homozygote-resistant males. It may be that heterozygote individuals are not affected in the same way by target site mutation and metabolic resistance driven by *GSTe2* enzymes. To confirm the lower mating ability of heterozygote mosquitoes compared to other genotypes as observed in this study, more work is needed in other locations to confirm such heterozygote disadvantage effect as the low sample size of L119F-RR homozygote resistant mosquitoes here could have impacted the assessment. Various studies conducted in other insect species on the impact of resistance on mating competitiveness showed that this trait of mosquitoes is not affected similarly. Resistant males displayed either a similar [e.g. *Metaseiulus occidentalis*: ^51^], a lower [(e.g. *Anopheles gambiae*: ^6^)] or a higher [(e.g. *Anopheles albimanus*: ^52^; *Tribolium castaneum*: ^53^] mating success when compared to the susceptible counterparts. Platt *et al*. (2015) also revealed an additive mating disadvantage in male homozygotes for both kdr/RDL-resistant alleles. However, because of the low frequency of RDL it was not possible to assess the cumulative impact of target site (RDL) with metabolic (GSTe2), although this could be interesting to do in the future in populations where both types of mechanisms co-exist.

It has previously been reported that metabolic resistance mechanisms, such as the overproduction of carboxylesterases as observed in resistant *C. pipiens*, could confer a significant fitness cost on mosquitoes life-traits. It was noticed in this species that resistant individuals displayed a reduced locomotive performance compared to the susceptible ones. It was suggested that such reduced performance was caused by a resource depletion linked to the overproduction of carboxylesterases ^54^. Prior to this study the only report of the impact of metabolic resistance on mating ability of malaria vector was conducted in 2015 in *An. gambiae.* Mating competitiveness in this species was not found to be significantly influenced by metabolic resistance mechanisms. However, that study ^10^ did not use a molecular marker for metabolic resistance, but a genome-wide microarray-based transcription analysis. The reduced performance of resistant mosquitoes in mating could contribute to slow the speed of increase in the frequency of resistant alleles in the wild, and will also prevent or delay the fixation of the resistance genes in the population. It is thus necessary that such studies are extended for other metabolic resistance mechanisms and in other locations with larger sample sizes in order to help implement successful management strategies.

## Conclusion

This study revealed a high and multiple resistance to insecticides, coupled with low efficacy of LLINs without PBO in *An. Funestus,* highlighting the threat that insecticide resistance poses on the efficacy of existing vector control tools. Interestingly, this study revealed that *An. funestus* swarms can be detected and characterized in the field providing the opportunity for mating swarms of this species to be targeted to implement alternative vector control strategies. Furthermore, this study provides preliminary evidences that metabolic resistance potentially exerts a fitness cost on mating competitiveness in resistant mosquitoes. As a negative fitness costs could influence the evolution of insecticide resistance in field populations of mosquitoes like the speed of increase or reversal to susceptibility in vector populations it is crucial that such impacts are understood and taken into consideration when designing and implementing future insecticide resistance management strategies.

## Data availability

### Underlying data

Underlying data is available from Open Science Framework

OSF: Dataset 1. Investigation of the influence of a glutathione S-transferase metabolic resistance to pyrethroids/DDT on mating competitiveness in males Anopheles funestus, African malaria vector https://doi.org/10.17605/OSF.IO/QD8P9 ^55^

Licence: CC0 1.0 Universal
