# Supplementary material for: Investigation of the influence of a glutathione S-transferase metabolic resistance to pyrethroids/DDT on mating competitiveness in males of the African malaria vector, Anopheles funestus
Source: Wellcome Open Res. 2019 Mar 21;4:13. Originally published 2019 Jan 24. [Version 2] doi: 10.12688/wellcomeopenres.15013.2 (PMC6480967; doi:10.12688/wellcomeopenres.15013.2)
Supplement: Peer review history

# Peer review: Reviewer response for version 2

**DOI:** 10.21956/wellcomeopenres.16580.r35095

**Published:** 2019-05-13

**Authors:** Jacques D. Charlwood

**Affiliations:** 1 Global Health and Tropical Medicine(GHTM), Institute of Hygiene and Tropical Medicine (IHMT), NOVA University Lisbon, Lisbon, Portugal

I have now read the revised version of the manuscript and think that it is suitable for indexing. I have no detailed comments to give you.

I have read this submission. I believe that I have an appropriate level of expertise to confirm that it is of an acceptable scientific standard.

# Peer review: Reviewer response for version 2

**DOI:** 10.21956/wellcomeopenres.16580.r35163

**Published:** 2019-04-17

**Authors:** Basil D. Brooke

**Affiliations:** 1 Centre for Emerging Zoonotic and Parasitic Diseases, National Institute for Communicable Diseases (NICD), National Health Laboratory Service (NHLS), Johannesburg, South Africa; 2 Wits Research Institute for Malaria, Faculty of Health Sciences, University of the Witwatersrand, Johannesburg, South Africa

This is an interesting manuscript that provides some comprehensive and potentially useful bionomic data for *Anopheles funestus* and, to a lesser extent, *An. gambiae* and *An. coluzzii* in Cameroon. My comments on its contents are as follows:

- The title does not adequately reflect the full scope of the content of this manuscript. Mating competitiveness is only one component of the data presented here.
- In the introduction (page 3) it is stated that little is known on the fitness costs of metabolic resistance mechanisms in anophelines owing to a lack of DNA based markers. I suggest that such markers, although of course useful, are not essential for such evaluations as fitness costs can also be assessed using biological characteristics such as life table analyses including fecundity and fertility. These likely give better indications of fitness cost. The authors should also think about what parameter they have actually measured here: was it mating competitiveness or propensity to mate? The former primarily speaks to physiological fitness while the latter is a behavioural issue. These terms may be interchangeable but there are subtle differences. Basically, less able to mate = reduced mating competitiveness; less inclined to mate = reduced propensity.  
- For the insecticide susceptibility assays, it should be noted that the standard method for assessing resistance intensity is by using increasing concentrations of the insecticide(s) in question. I assume the authors chose to use extended exposure times instead because of a shortage of test samples (F1s) but the problem with this method is that there is no clearly elucidated method for assessing the operational implications of increased intensity where detected using data generated in this way.
- The data on mating competitiveness are somewhat ambivalent because the genotype frequencies for the L119F-GSTe2 resistant homozygotes (RR) were equivalent between mated and unmated males. It is therefore difficult to see how this mutation negatively impacts mating competitiveness/propensity. As the only real difference was the frequency of heterozygotes in each group, an alternative explanation may point to an associative negative heterotic effect on propensity to mate in males that is not caused by the L119F-GSTe2 mutation itself, but rather by linkage disequilibrium between this locus and other deleterious alleles i.e. a negative pleiotropic effect.

Minor corrections:

- ‘Anopheles’ should be italicised throughout.
- Is ‘Koekemoer cocktail Polymerase Chain Reaction assay’ an official name for this assay?

I have read this submission. I believe that I have an appropriate level of expertise to confirm that it is of an acceptable scientific standard, however I have significant reservations, as outlined above.

# Peer review: Reviewer response for version 2

**DOI:** 10.21956/wellcomeopenres.16580.r35094

**Published:** 2019-03-29

**Authors:** Adriana E. Flores

**Affiliations:** 1 Facultad de Ciencias Biológicas, Universidad Autónoma de Nuevo León, San Nicolás de los Garza, Mexico

The new version of the article is acceptable as it is.

I have read this submission. I believe that I have an appropriate level of expertise to confirm that it is of an acceptable scientific standard.

# Peer review: Reviewer response for version 1

**DOI:** 10.21956/wellcomeopenres.16378.r34761

**Published:** 2019-02-20

**Authors:** Adriana E. Flores

**Affiliations:** 1 Facultad de Ciencias Biológicas, Universidad Autónoma de Nuevo León, San Nicolás de los Garza, Mexico

This manuscript represents the evaluation of the male fitness of *Anopheles funestus* in relation with mating competitiveness with different levels of insecticide resistance to permethrin, deltamethrin and DDT, and mechanisms involved.

 The authors established the presence of CyP450 and GSTs with the use of synergists. They also determined the relationship between the L119F-GSTe2 metabolic resistance and the impact on the mating success besides the frequency of L1014F kdr mutation in the populations analyzed.

 Overall the study is complete, well organized, and the analysis clearly shows the influence of the presence of the resistant allele for GSTe2 on the mating competitiveness. On the other hand, the authors found a high frequency of 1014F kdr resistance allele in the populations analyzed. I wonder, why the authors did not consider kdr resistance altogether with GSTe2. Either way, both mechanisms are present in the population. Could there be an interaction of the presence of both resistant alleles (kdr and GSTe2) in relation to the mating competition?

I have read this submission. I believe that I have an appropriate level of expertise to confirm that it is of an acceptable scientific standard, however I have significant reservations, as outlined above.

# Response

**Published:** 2019-03-13

**Authors:** Magellan TCHOUAKUI

**Affiliations:** Centre for Research in Infectious Diseases, Cameroon

We really appreciate this comment from the reviewer. We just want to highlight that a high frequency of 1014F kdr resistance allele found was for *An. gambiae* mosquitoes since for instance, there is no evidence of *Kdr* in *An. funestus*. For this reason, we could not assess the interaction of the presence of *kdr* and *GSTe2* in relation to the mating competition in *An funestus*. However, as the *RDL* and *GSTe2* are both present in this species we wanted to assess the cumulative impact of these genes on mating competition but this was not possible because of the very low frequency of the A296S-RDL mutation in this *An. funestus* population.

# Peer review: Reviewer response for version 1

**DOI:** 10.21956/wellcomeopenres.16378.r34652

**Published:** 2019-02-11

**Authors:** Jacques D. Charlwood

**Affiliations:** 1 Global Health and Tropical Medicine(GHTM), Institute of Hygiene and Tropical Medicine (IHMT), NOVA University Lisbon, Lisbon, Portugal

Investigation of the influence of a glutathione S-transferase metabolic resistance to pyrethroids/DDT on mating competitiveness in males Anopheles funestus, African malaria vector.

 The title of this paper is somewhat of a misnomer.  It is probably described thus to attract a wider audience than a more somewhat mundane title describing the resistance status of the Anopheles funestus from a village in Cameroon. The paper describes in considerable detail the resistance status of the mosquito more than it does the effect of this on mating success. Thus, the results concerning mating success concern just 21 males caught in copula whilst the overall resistance status covers a number of tests from standard WHO tests to tests with the synergist PBO and bioassays on different net types.

 The authors discuss the fact that results on mating competitiveness of resistant mosquitoes from a small number of studies give confusing results. Indeed they have participated in studies where five times the number of mating mosquitoes have been investigated.  Some indicate that there may actually be an advantage whilst others give different results. It obviously is a field that can be explored further. But in the present case I feel that the authors are confusing statistical significance with biological significance. They do point out that further studies would be useful but given their title,which others may take for a fact, this is a problem.

 Given that the swarms the authors observed occurred close to houses it would seem that the males rested inside houses (as they do elsewhere). It is a shame that they did not examine the proportions of the resistance genotype among these insects. Indeed, it might have been possible to collect resting insects and to examine their terminalia to determine the degree of rotation so that the effect of age on survival and resistance status among the males could have been investigated.

 The authors write ‘Concerning the mating behaviour, when a female chose a male, they immediately left the swarms, flying at 1.5m from the ground.’ But it is by no means certain that female mosquitoes ‘choose’ their mates (which implies that sexual selection is taking place). With respect to their earlier paper it is perhaps worth pointing out that (as far as I know) there is no evidence of olfaction playing a part in the mating behaviour of Anopheles mosquitoes.

 Given that the swarms seen were large (more than 100 individual males in a swarm) it begs the question as to how many mating pairs were seen and how many were successfully collected. If after 12 nights of observation of such large swarms only 21 pairs were formed it begs the question ‘Is this all the mating that was taking place?’  Either there were other sites where a lot more mating was taking place or something else is going on. I do not know.

 The place used by the mosquitoes to swarm is similar to that described from Mozambique. There the mosquitoes actively avoid markers if they are introduced under the swarm. Again the characteristics of the location remain undefined – why they swarm where they swarm is still an enigma but as the authors point the insects remain consistent and swarm in the same place night after night.

 Given the time and effort that resistance tests require their extensive data on resistance should allow them to suggest what is the optimum or minimal method that could be used to determine the resistance status in other populations.  This is perhaps a better focus for their paper since much of the information that they provide is irrelevant to the title of the paper. If the paper continues with the same theme then much of the data that they present could, in fact be provided as supplemental files.

 Given the advantage conferred by resistance alleles over susceptible ones and given the very high level of resistance it is strange that these genes are not fixed in the population. It also means that if in the absence of insecticide pressure resistant insects are at a disadvantage compared to the wild type (perhaps because they have to divert resources from eggs to cuticle) the presence of susceptible insects implies that once insecticide pressure is removed the population will revert back. How this might affect transmission is moot. 

 The English in the paper could be improved. For example, in my opinion, the very first sentence reads better thus: 'Despite an increase in the last two years1 significant progress has been made in recent decades in malaria vector control. This has contributed to a significant reduction in the burden of disease caused by this parasite'. Additionally, if the title if it is retained, can be rearranged thus ‘Investigation of the influence of a glutathione S-transferase metabolic resistance to pyrethroids/DDT on mating competitiveness in males of the African malaria vector, *Anopheles funestus*’

 It is also a bit of a shame that the sporozoite data for the *Anopheles gambiae* s.l. were not given by species. With such a high rate both members of the complex would be expected to be infected. If only one species was then this would not only boost the rate – to very high levels – but would also indicate that there might be differences in vectorial capacity between the species that merit further investigation. It would seem reasonable to assume that they were dealing with ageing populations in decline after the rainy season which was responsible for the high sporozoite rates.

 Presumably the A. funestus could be controlled (for the time being at least) by indoor residual spray of an insecticide like, the current flavor of the month, primiphos-methyl (Actellic).

I have read this submission. I believe that I have an appropriate level of expertise to confirm that it is of an acceptable scientific standard, however I have significant reservations, as outlined above.

# Response

**Published:** 2019-03-13

**Authors:** Magellan TCHOUAKUI

**Affiliations:** Centre for Research in Infectious Diseases, Cameroon

**Comment 1: **The title of this paper is somewhat of a misnomer. It is probably described thus to attract a wider audience than a more somewhat mundane title describing the resistance status of the *Anopheles funestus* from a village in Cameroon. The paper describes in considerable detail the resistance status of the mosquito more than it does the effect of this on mating success. Thus, the results concerning mating success concern just 21 males caught in copula whilst the overall resistance status covers a number of tests from standard WHO tests to tests with the synergist PBO and bioassays on different net types.

**Response: **We sincerely appreciate this comment from the reviewer. The main aim of this study was to evaluate the impact of insecticide resistance on mating competition using the L119F-GSTe2 resistance marker hence the title: ‘’Investigation of the influence of a glutathione S-transferase metabolic resistance to pyrethroids/DDT on mating competitiveness in males *Anopheles funestus*, African malaria vector’’. Because prior to this study the resistance profile of malaria vectors in the locality was unknown, it was important to start by elucidating the resistance profile and the potential mechanisms driving the resistance to insecticide in the study site. So the title was not chosen to attract a wider audience as the reviewer is noticing but refers to the main question we wanted to address when designing this study. Also by using the word “investigation” in the title, we are acknowledging that more work is needed to fully establish this impact on mating but this provides initial useful observations on this topic.

**Comment 2: **The authors discuss the fact that results on mating competitiveness of resistant mosquitoes from a small number of studies give confusing results. Indeed they have participated in studies where five times the number of mating mosquitoes have been investigated. Some indicate that there may actually be an advantage whilst others give different results. It obviously is a field that can be explored further. But in the present case I feel that the authors are confusing statistical significance with biological significance. They do point out that further studies would be useful but given their title, which others may take for a fact, this is a problem.

**Response: **We thank again the reviewer. We agree that there is a risk of confusing statistical significance with biological significance. But we believe that we have avoided this situation by presenting each result as we saw it. We noticed when using the Odd-ratio comparison that mosquitoes with the 119F resistant allele displayed significantly lower mating competitiveness compared to those with the L119 susceptible allele and we provided the statistical significance associated with the observation. Then we discussed the potential meaning of the result but taking a cautionary approach by stating that although it could mean that there is a biological meaning that only further studies will confirm this. This is the general message of this manuscript and we agree with the reviewer that such confusion should be avoided that is why we mentioned in discussion that further studies are needed in other locations and other resistant markers to widely appreciate the impact of resistance on mating competitiveness.

**Comment 3: **Given that the swarms the authors observed occurred close to houses it would seem that the males rested inside houses (as they do elsewhere). It is a shame that they did not examine the proportions of the resistance genotype among these insects. Indeed, it might have been possible to collect resting insects and to examine their terminalia to determine the degree of rotation so that the effect of age on survival and resistance status among the males could have been investigated.

**Response:** We thank the reviewer for noting this. Our main aim as mentioned above was mainly to see if insecticide resistance has any impact on mating competition through swarm collection. That is why we did not collect resting males to examine their terminalia and determine the degree of rotation as pointed by the editor but this is a good idea that will be taken into consideration in future studies.

**Comment 4: **The authors write ‘Concerning the mating behaviour, when a female chose a male, they immediately left the swarms, flying at 1.5m from the ground.’ But it is by no means certain that female mosquitoes ‘choose’ their mates (which implies that sexual selection is taking place). With respect to their earlier paper it is perhaps worth pointing out that (as far as I know) there is no evidence of olfaction playing a part in the mating behaviour of Anopheles mosquitoes.

**Response: **We agree with the reviewer that there is very little information on the impact of olfaction on the mating behavior of Anopheles mosquitoes but in this study we noticed that when a female coupled with a male, they immediately left the swarms, flying at 1.5m from the ground for copulation although the mechanisms involved remain unknown. To avoid confusion we have replaced “chose” by “coupled with”.

**Comment 5: **Given that the swarms seen were large (more than 100 individual males in a swarm) it begs the question as to how many mating pairs were seen and how many were successfully collected. If after 12 nights of observation of such large swarms only 21 pairs were formed it begs the question ‘Is this all the mating that was taking place?’  Either there were other sites where a lot more mating was taking place or something else is going on. I do not know.

**Response:** It is true that given the size of the swarms 21 couples collected were very low but this can be explained by the fact that females are not necessarily participating to the swarming; that is why the number of unmated males was very high. But the hypothesis that this was not all the mating taking place cannot be rejected since when the night started it was not easy to observe the couples. We have now added this sentence to highlight it: “The low number of collected couples suggests a low number of females in these swarms but could also indicate that mating was also taking place in other swarms not detected in this study.”

**Comment 6: **The place used by the mosquitoes to swarm is similar to that described from Mozambique. There the mosquitoes actively avoid markers if they are introduced under the swarm. Again the characteristics of the location remain undefined – why they swarm where they swarm is still an enigma but as the authors point the insects remain consistent and swarm in the same place night after night.

**Response: **We appreciate this comment from the reviewer. Compared to *An. gambiae* complex which swarm around markers such as brick piles, rice fields, banana trees, burnt ground, garbage heaps and ant hills *An. funestus* swarms we observed in this study appeared to avoid ground markers. So it will be important in the future to assess the ecological and physiological parameters enhancing *An. funestus* mating.

**Comment 7: **Given the time and effort that resistance tests require their extensive data on resistance should allow them to suggest what is the optimum or minimal method that could be used to determine the resistance status in other populations.  This is perhaps a better focus for their paper since much of the information that they provide is irrelevant to the title of the paper. If the paper continues with the same theme then much of the data that they present could, in fact be provided as supplemental files.

**Response: **Thank you for your comments. We cannot suggest what is the optimum or minimal method that could be used to determine the resistance status in other populations since there are WHO’s recommendations on how to assess the resistance profile in a given population and we were just following those instructions. We suggest keeping the whole data as it is as we explained above the insecticide work was necessary in order to explore the impact of the GSTe2 resistance allele on mating competition.

**Comment 8: **Given the advantage conferred by resistance alleles over susceptible ones and given the very high level of resistance it is strange that these genes are not fixed in the population. It also means that if in the absence of insecticide pressure resistant insects are at a disadvantage compared to the wild type (perhaps because they have to divert resources from eggs to cuticle) the presence of susceptible insects implies that once insecticide pressure is removed the population will revert back. How this might affect transmission is moot. 

**Response: **Thanks to the reviewer for this remark. Some resistance genes are fixed in mosquito’s populations like *Cy6p9a/b* in Southern Africa, the L119F-GSTe2 in Benin for *An. funestus* and the L1014F-Kdr in many African *An. gambiae* populations. However fixation of resistance alleles depends on other factors and the mode of selection of these alleles. That is why to it is crucial to study the impact of resistance on mosquito’s life such mating competitiveness before implementing any resistance management strategy based on rotation because when the resistance becomes fixed in the population there is little chance to revert to susceptibility.

**Comment 9: **The English in the paper could be improved. For example, in my opinion, the very first sentence reads better thus: 'Despite an increase in the last two years1 significant progress has been made in recent decades in malaria vector control. This has contributed to a significant reduction in the burden of disease caused by this parasite'. Additionally, if the title if it is retained, can be rearranged thus ‘Investigation of the influence of a glutathione S-transferase metabolic resistance to pyrethroids/DDT on mating competitiveness in males of the African malaria vector, *Anopheles funestus*’

**Response: **We have double-checked the whole manuscript for English grammar and mistakes.

**Comment 10: **It is also a bit of a shame that the sporozoite data for the *Anopheles gambiae* s.l. were not given by species. With such a high rate both members of the complex would be expected to be infected. If only one species was then this would not only boost the rate – to very high levels – but would also indicate that there might be differences in vectorial capacity between the species that merit further investigation. It would seem reasonable to assume that they were dealing with ageing populations in decline after the rainy season which was responsible for the high sporozoite rates.

**Response: **We combined the Plasmodium infection rate in *An. gambiae s.l* since *An. coluzzii *represented only 17.5% ( *An. gambiae* (n= 33/40) and *An. coluzzii* (n=7/40)) of these mosquitoes. However, among the 5 mosquitoes infected with Plasmodium sporozoites2 were *An. coluzzii* [2/7 infected (28.5%)] and 3 were *An. gambiae* [3/33 infected 9.1%)]. However, the low sample size of *An. coluzzii* means that this rate is not comparable. Nevertheless we have now presented the infection rate for each species in the manuscript.

**Comment 11: **Presumably the *A. funestus* could be controlled (for the time being at least) by indoor residual spray of an insecticide like, the current flavor of the month, primiphos-methyl (Actellic).

**Response: **We sincerely appreciate this suggestion from the reviewer. It is true that this insecticide could be used to control this *An. funestus* population since a full susceptibility was observed for organophosphate insecticides in WHO tube assays.
